# Evaluating Disease Patterns of Military Working Dogs in the Republic of Korea: A Retrospective Study

**DOI:** 10.3390/ani13081400

**Published:** 2023-04-19

**Authors:** Sanghyeon Park, Gyeonggook Park, Mi-Sun Rieu, Taewoo Kim, Dongwook Kim, Sungin Lee, Gonhyung Kim

**Affiliations:** 1 Department of Veterinary Surgery, College of Veterinary Medicine, Chungbuk National University, Cheongju 28644, Republic of Korea; 2 Department of Veterinary Medicine, Armed Forces Medical Research Institute, Daejeon 34059, Republic of Korea

**Keywords:** military working dogs, gastrointestinal disease, foreign body, vector-borne pathogens

## Abstract

**Simple Summary:**

Evaluating disease patterns of military working dogs is important for effective preventive care. The disease patterns may be affected by breed and living and working conditions. This study reviewed medical data from the Armed Forces Medical Research Institute in the Republic of Korea. Gastrointestinal diseases due to foreign body ingestion were the most common. To prevent ingestive behavior disorders, such as foreign body ingestion, it is important to evaluate and improve the environment of military working dogs that may induce stress. Surgery for dental disease or the removal of a gastric foreign body was generally performed. Therefore, preventive care for dental disease and foreign body ingestion may be helpful for the effective performance and good quality of life in Korean military working dogs.

**Abstract:**

The purpose of this study was to evaluate disease patterns among military working dogs (MWDs) at the Armed Forces Medical Research Institute (AFMRI) to provide basic medical data on Korean MWDs. The medical records of procedures performed at AFMRI between November 2017 and March 2021 were reviewed. Physical examination, diagnostic imaging, and laboratory tests were performed according to the status of each dog. A total of 353 MWDs (215 males and 138 females; mean age, 6 ± 3 years) were analyzed in this study. Among Korean MWDs, gastrointestinal (GI) disorders are the most frequently diagnosed, followed by dental and musculoskeletal disorders. Foreign body (FB) ingestion had the highest prevalence of GI disorders, with the most common FB being a leather collar or leash. General and dental surgeries, including removal of gastric FB and tooth extraction, were routinely performed at the AFMRI. Preventative care focusing on dental disease and FB ingestion may be helpful for effective performance and good quality of life in MWDs, with the regular assessment and prevention of environmental factors that may contribute to behavioral problems such as FB ingestion, coprophagy, and anorexia.

## 1. Introduction

The Republic of Korea (ROK) maintains military working dogs (MWDs) for guarding, scouting, and explosive detection. The Korean MWDs mainly consist of the following breeds: German Shepherd Dog (GSD), Belgian Malinois (BM), Labrador Retriever (LR), and a small number of English Springer Spaniels (ESS). Korean MWDs are born and raised during the puppy stage, receiving training to adapt to the military environment (people, other MWDs, noise, vehicle rides, etc.) and form a relationship with their handlers. MWDs live in kennels within the military unit. Most kennels are spacious and well-maintained. These dogs undergo various types of training, including obstacle, boundary, aggression control, and scouting/searching training. They spend several hours a day performing their duties and undergoing training, while also receiving adequate rest periods.

The breeding and training corps of MWDs include the MWD training center and the Republic of Korea Air Force (ROKAF) education and training command, each of which has a veterinary hospital for MWDs. Several Korean veterinary and non-commissioned officers and civilian workers in the military are responsible for the veterinary care of MWDs. The Armed Forces Medical Research Institute (AFMRI) under the Armed Forces Medical Command provides veterinary care for MWDs.

Compared to other veterinary hospitals, military veterinary hospitals are specific to the dog breeds mentioned above. The utility and longevity of MWDs are presumably affected by diseases that are common to the specific sizes, breeds, and functions of MWDs [1]. Many studies have been conducted on diseases in specific dog breeds [2,3,4,5,6]. For example, one study analyzed disease patterns in 32,486 GSDs, which constitute the majority of Korean MWDs [5]. In that study, locomotor disorders were the most common cause of death in GSDs.

In a previous study on guide dogs, musculoskeletal conditions (primarily arthritis) were the most common reason for retirement, accounting for 28% (387/1362) of cases. Skin conditions, such as atopic dermatitis, and nervous sensory conditions, including epilepsy (35% of cases), were also identified as causes for reduced working life [7]. In a 2017 study by Shaffer on dogs employed in law enforcement and search and rescue, mutational analysis was conducted, revealing the genetic risks of exercise-induced collapse, degenerative myelopathy, and leukocyte adhesion deficiency. The study suggests performing genetic screening before breeding, purchasing, or training dogs to prevent conditions that can result in emotional costs for handlers and financial costs for service organizations due to the loss of dogs caused by early retirement or euthanasia [8].

Various studies have been carried out on disease patterns in working dogs [1,7,8,9,10,11,12,13,14,15]. However, the disease patterns of Korean MWDs patients have not yet been reported. In a previous study on the cause of death or euthanasia of MWDs [1], the leading causes were appendicular degenerative joint disease (DJD), neoplasia, spinal cord disease, nonspecific geriatric decline, and gastric dilatation-volvulus (GDV).

Military veterinarians should be aware of the diseases that the MWDs are predisposed to. The effective preventive care of working dogs not only requires standard procedures applicable to dogs, but also more specific ones based on their characteristics of breed, geographic location, and living and working conditions [9].

Comprehensive preventive care is needed for working dogs to help them perform their best duties properly [9]. Evaluating the disease patterns of Korean MWDs may provide information on the prevention and treatment of diseases in Korean MWDs. The purpose of this study was to evaluate disease patterns among MWDs of AFMRI to provide basic medical data on Korean MWDs for effective preventive care.

## 2. Materials and Methods

### 2.1. Study Population

The study population consisted of 353 MWDs who presented at the AFMRI between November 2017 and March 2021. In total, 1072 medical cases of these 353 MWDs were examined. Age groups were classified into young adult (1–4 years), mature adult (5–9 years), senior (10–12 years), and end-of-life (over 13 years) based on the 2019 American Animal Hospital Association Canine Life Stage Guidelines [16].

The puppy stage was excluded because AFMRI only had records of adult dogs over 1 year of age. The end-of-life stage was excluded from the study because the number of cases in this group was less than that in the other groups, and most of the cases were of medical examination during retirement. ESSs were excluded because they are not large breeds similar to others, and would be gradually excluded from MWDs.

### 2.2. Medical Records

All medical records, including electronic medical records and medical charts written by hand or word processors, that were available at the time of research were analyzed. Medical data included breed, duty, sex, age, body weight, clinical signs, diagnosed diseases, and type of surgery. Physical examination, diagnostic imaging, and laboratory tests were performed according to the status of each dog. Data duplication was minimized by analyzing only the first diagnosis. Cases of same dogs on different dates and pathological causes were classified into different cases. Through the overall evaluation, one chief concern and one disease category were selected to describe the patient’s current problem. Differential (tentative) or definitive diagnoses were classified into categories used by AFMRI.

Cases of specific examination or surgical procedures for secondary care requested by veterinary officers in the MWD operating unit and regular medical examination without specific chief concerns were excluded from the analysis of causes of veterinary care in MWDs. Cases with a category of no remarkable findings or unknown causes were excluded from the analysis of disease categories in MWDs. The seasons were categorized as spring (March, April, and May), summer (June, July, and August), fall (September, October, and November), and winter (December, January, and February).

SNAP^®^ 4Dx^®^ (IDEXX Laboratories, Inc., Westbrook, ME, USA) was used for the detection of antibodies of *Anaplasma* spp. (*A. phagocytophilum* and *A. platys*), *Borrelia burgdorferi*, and *Ehrlichia* spp. (*E. canis*, and *E. ewingii*), and antigens of *Dirofilaria immitis*.

In analyzing cases of surgical procedures, performing more than two procedures on one dog was counted as a separate case. Prophylactic procedures, including dental prophylaxis, elective neutering, and prophylactic gastropexy, were excluded.

MWDs with gait abnormality as a chief concern or presenting with lameness on gait examination were included in the analysis of gait abnormality. Cases with normal gait patterns during the examination or only tentative diagnoses were excluded.

The medical records of the foreign body (FB) included the type of foreign object, location, and removal method: upper gastrointestinal (GI) endoscopy, gastrotomy, enterotomy, and spontaneous evacuation. Cases with no specific data on the types of FB were excluded from the analysis of types of foreign objects.

## 3. Results

In this study, the most common duty of the MWDs was guarding, followed by scouting and explosive detection. GSD was the most common breed, followed by BM, LR, and ESS (Table 1). Intact male dogs were the most common, followed by spayed females, castrated males, and intact females. In terms of age groups, young adults were the most common, followed by mature adults, seniors, and end-of-life patients. The mean age of the patients was 6 ± 3 years (mean ± standard deviation).

MWDs were most commonly diagnosed with GI diseases (mainly FB ingestion), followed by dental (mainly periodontal disease or tooth fracture) and musculoskeletal diseases (Table 2). GI diseases were the most common issues in both GSD and BM, while dental and ear/skin diseases were frequently diagnosed in LR (Table 3). GI diseases were also more common in young and mature adult dogs, while neurological and oncological diseases were prevalent in senior dogs (Table 4). Regardless of the season, GI diseases were the most commonly diagnosed conditions (Table 5).

The SNAP^®^ 4Dx^®^ test revealed that *Anaplasma* spp. were the most abundant in MWDs, followed by *D. immitis*, *Ehrlichia* spp., *B. burgdorferi* with *Ehrlichia* spp., *Anaplasma* spp. with *Ehrlichia* spp., *Anaplasma* spp. with *B. burgdorferi*, and *B. burgdorferi* (Table 6).

In AFMRI, dental surgeries were the most commonly performed, followed by GI surgeries. Dental scaling for periodontal diseases was the most common surgery, followed by tooth extraction and upper GI endoscopy or gastrotomy for FB removal. Castration for the treatment of suspected benign prostatic hyperplasia, cryptorchidism and orchitis, dental resin restoration for vital pulp therapy or dental defects, repair of skin wound, excision of the gingival mass, and splenectomy were commonly performed (Table 7).

Musculoskeletal diseases, including DJD, sprain/strain, and cranial cruciate ligament (CrCL) disease, were the most commonly diagnosed diseases in MWDs with gait abnormality, followed by neurological diseases, including intervertebral disc disease (IVDD) and degenerative lumbosacral stenosis (DLSS) (Table 8).

The foreign objects found were leather collars or leashes, metallic objects, towels or gloves, plastic objects, stones and sand, balls, string, one oxygen absorber used in the packages of dog snacks, fruit seeds, and electric wires (Table 9). Most foreign objects were found in the stomach, followed by the small intestine, large intestine, and esophagus. Foreign objects were most commonly removed via upper GI endoscopy, followed by gastrotomy, spontaneous evacuation, and enterotomy (Table 10).

## 4. Discussion

In the ROK, there are three major military veterinary hospitals (MWD training center, ROKAF education and training command, and AFMRI). MWDs in need of veterinary care visit military veterinary hospitals at the request of their handlers or veterinary officers in the MWD operating unit. Some MWD operating units have veterinary officers for primary care, whereas others do not. MWDs located far away from military veterinary hospitals may find it difficult to receive immediate veterinary care. Therefore, regular medical examinations of MWDs are essential. Comprehensive, but specific, preventive care for MWDs based on disease patterns is vital. Military veterinarians play a major role in the duties performed by MWDs, as well as in their health and welfare.

Although gait abnormality was a common cause of veterinary care of MWDs at the AFMRI, ingestion of FB and clinical signs of GI disorders, including diarrhea, vomiting, and hematochezia, were even more prevalent. Cases of gait abnormality and FB ingestion were analyzed for the further evaluation of surgical diseases. In a previous study, the most frequently recorded disorders in 3884 dogs were otitis externa, periodontal disease, and anal sac impaction [17]. In this study, the most common disorder was GI diseases, followed by dental and musculoskeletal diseases. Gastric FB had the highest prevalence of GI diseases, followed by suspected gastroenteritis with varying severity and inflammatory bowel disease, which GSD were found to have a higher risk for [18]. Tooth fractures and periodontal diseases were the most common dental diseases. Traumatic dental injuries, including tooth fractures, can result from a variety of factors such as training, housing or environmental conditions, and behavior [19,20]. While the number of cases evaluating the causes of dental injuries in the medical records of AFMRI was limited, tooth fractures in Korean MWDs may be also related to a similar etiology. DJD was the most common musculoskeletal disease, followed by sprains and strains. These are the major causes of gait abnormalities that will be described later.

In a previous study examining disease patterns in 32,486 GSDs, skin disorders were found to be the most frequent disease category (morbidity) [5]. However, in the present study, GI disorders were the most prevalent, with the number of cases being 2.3 times higher than the combined cases of ear and skin disorders. BM also had a high prevalence of GI diseases. In contrast, for LR, ear/skin disorders and dental diseases were the most frequently diagnosed issues, although the number of cases was relatively small. A previous study on LRs found otitis externa to be the most prevalent disease (prevalence 10.4%, 95% confidence interval: 9.1–11.8) [21]. Further investigation is needed to determine if ear/skin disorders or dental diseases in MWDs are more common in LR compared to other breeds.

Both young and mature adult dogs were frequently diagnosed with GI, dental, and ear/skin diseases. However, senior dogs had a higher incidence of neurological and oncological diseases compared to GI diseases. The high prevalence of neurological diseases in senior dogs is believed to be caused by degenerative conditions such as DLSS and IVDD, which are common in GSDs, the majority breed of MWDs in Korea [22,23]. Oncological diseases were also frequently observed in senior dogs due to their higher incidence in later stages of life [16,24]. In Korean MWDs, GI diseases were the most commonly diagnosed condition throughout all four seasons, with no significant difference in incidence by season.

Tick-borne pathogens can cause various diseases, such as anaplasmosis, ehrlichiosis, and Lyme disease, in dogs. Recently, the incidence of tick-borne diseases, such as severe fever with thrombocytopenia syndrome, Lyme disease, and Q fever, in humans has also increased rapidly in the ROK [25]. In a study of 532 outdoor dogs excluding MWDs, a serological survey using the IDEXX SNAP^®^ 4Dx^®^ test revealed the highest prevalence of *D. immitis* (25.2%), followed by *A. phagocytophilum* (15.6%) and *E. canis* (4.7%), while *B. burgdorferi* showed the lowest prevalence (1.1%) [26]. In the present study, *Anaplasma* spp. was the most abundant, followed by *D. immitis* and *Ehrlichia* spp. The seroprevalence of *Anaplasma* spp., *B. burgdorferi* sensu lato, and *Ehrlichia* spp. was significantly higher in MWDs (*n* = 308) than in companion (*n* = 938) or shelter dogs (*n* = 969) in another study [27].

Identification of DNA evidence of active infections in MWDs with a positive SNAP^®^ 4Dx^®^ test was not possible in this study. In a study of MWDs at United States military locations in the ROK, no significant differences were found in the seroprevalence of tick-borne infections based on location, year, breed, or sex of the MWD [13]. Further studies on the risk factors of vector-borne diseases in Korean MWDs, including the environment of duty performance, are necessary. Preventive care for vector-borne diseases in dogs and humans in the military is essential.

Except for prophylactic procedures, dental surgeries were most commonly performed in AFMRI due to the high prevalence of dental diseases. Working dogs have a similar prevalence of periodontal disease as companion dogs, and untreated periodontal disease can cause local and systemic effects [19]. Routine dental prophylaxis for MWDs is vital because the oral health of MWDs is essential to scent detection and overall health. Primary dental care for MWDs by their handlers is also important.

Following surgery for the removal of the FB, castration for the treatment of male reproductive diseases such as benign prostatic hyperplasia and cryptorchidism was commonly performed. In this study population, intact males were more common than castrated males. Some handlers believe that castration reduces the ability of male dogs to perform their duties. However, in a previous study on Swiss military dogs, chemically castrated males performed their duties as well as intact males [28]. Castrated or spayed dogs have a longer lifespan than intact dogs, although neoplasia is more frequent at later ages [1,16,24,29]. In a previous study, castrated male dogs were significantly more likely to have hip dysplasia than other dogs, and castrated or spayed dogs were more likely to have CrCL deficiencies [30]. In another study on the neutering of GSDs, increased joint disorders with CrCL tears was associated with early neutering before 12 months [6]. Further studies are needed to determine the appropriate timing for neutering to reduce the prevalence of both musculoskeletal and reproductive disorders, which can affect the performance and lifespan of MWDs.

Gait abnormality is a critical clinical sign affecting MWDs, which reduces their effective performance of duties and quality of life. Large breeds such as GSD, LR, and BM are predisposed to locomotor disorders such as elbow and hip dysplasia, and spinal cord disease [1,2,4,5,9,30,31]. The major cause of death or euthanasia in MWDs (927 cases) was appendicular DJD in one study [1]. In this study, the most definitive diagnosis of MWDs with gait abnormality was musculoskeletal diseases, such as DJD. Approximately 77% of DJD cases were associated with the hip joint. Some dog breeds that comprise Korean MWDs have been reported to have a high prevalence of hip dysplasia [6,30]. A previous study found that the prevalence of hip dysplasia in purebred dogs was 19.7%, while the prevalence in mixed breed dogs was 17.7% [32]. The probability of hip DJD increases with hip joint laxity and an increase in age in some large breeds including GSD and LR [2]. For effective performance and quality of life, MWDs with genetic factors of hip dysplasia should be excluded from breeding, and the military should routinely monitor for hip joint disorders through further research.

Gait abnormalities in MWDs are frequently diagnosed as acute sprains or strains. Acute injuries to the locomotor system can occur during physical activities such as training, working, and playing. Furthermore, the chronic workload may exacerbate the lameness of MWDs with DJD. Therefore, it is crucial to prevent injuries during work or training through measures such as muscle warming, joint protection, appropriate rest, and recovery.

Of the 29 MWDs with neurological dysfunctions such as paraparesis or general proprioceptive ataxia, only 10 dogs were evaluated by magnetic resonance imaging (MRI) at other university veterinary hospitals, because diagnosis using MRI was not available at the AFMRI. The early detection and treatment of neurological diseases such as IVDD and DLSS are vital for increased working lifespan and good quality of life for MWDs [22]. Therefore, it is essential to ensure that the MWDs can be evaluated using MRI in military veterinary hospitals.

The ingestion of foreign objects is a common diagnosis, and one of the leading causes of emergency veterinary care [33,34,35,36]. Nevertheless, one study indicated that there were no reports of an apparent underlying cause of FB ingestion [37], which is primarily of a behavioral nature (88% of cases) rather than digestive pain (12% of cases), and the regular shredding of objects is related to hyperactivity–impulsivity disorder, whereas its absence was related to anxiety or attachment disorder in a previous study [38]. In the present study, 9 of the 42 MWDs ingested FB more than twice. A comprehensive evaluation of behavioral problems should be conducted on MWDs with a history of FB ingestion, which may be important because the most common cause of discharge of MWDs aged under 5 years was behavioral problems (82.3%) in another study [12].

The prevalence of ingestive behavior disorders was difficult to determine in this study due to the limited availability of behavioral evaluation records that could assess symptoms such as FB ingestion, anorexia, and coprophagy induced by stress. In a previous study on behavioral problems, the prevalence of pica was found to be 34.9% (715/2050) [39]. The odds of pica were higher in young dogs compared to adult dogs and in neutered dogs compared to intact dogs. Similar to the study, the prevalence of FB ingestion was also highest among young adults (51.9%, 27/52), followed by mature adults (30.8%, 16/52), and seniors (17.3%, 9/52). Neutered dogs (76.9%, 40/52) had a higher incidence of FB ingestion compared to intact dogs (23.1%, 12/52). A previous study on coprophagy found that about 12.9% of shelter dogs adopted within 4 weeks showed coprophagy [40]. In another previous study using a web-based survey, it was found that 23% of dogs had eaten feces at least once, and 16% had eaten feces at least six times [41].

Stress-induced issues have been linked to ingestive behavioral problems, including FB ingestion, anorexia, hyperphagia, and coprophagy [42,43,44,45]. While medical records from AFMRI contain well-documented cases of foreign object ingestion, there is a lack of medical records recognizing eating behavioral problems as stress-related disorders. Many coprophagic MWDs have also been observed, but no investigations have been conducted on all MWDs. The high prevalence of FB ingestion in this study may be related to stress from various factors, such as living and working environments and relationships with handlers.

While most of the living conditions for Korean MWDs are spacious, comfortable, and well-managed, this may not be the case for all units. Previous studies have shown that human interaction and environmental enrichment can reduce stress in dogs living in kennels, such as laboratory dogs, shelter dogs, and working dogs [43,46,47]. Maintaining a positive relationship with handlers and improving the kennel environment may help prevent eating behavioral disorders.

Various foreign objects have been ingested by dogs in many studies [36,38,48]. In this study, leather collars or leashes were the most common FB among the examined cases, followed by metallic objects, towels or gloves, and plastic objects, which could be easily found near the cages of MWDs. Foreign objects were found most commonly in the stomach and were removed mainly by endoscopy. In a previous study, the endoscopic removal of gastric FB was associated with a high success rate [48]. In one case in this study, a long leather leash acted as a linear FB and caused intussusception of the small intestine, which was removed using two enterotomies. Linear FB produces more severe clinical signs, such as vomiting, anorexia, lethargy, or abdominal pain, and a greater risk for intestinal perforation than non-linear FB [33,34]. To prevent MWDs from ingesting foreign objects, it is essential to assess the environmental and workload factors that cause stress in dogs, and the resultant behavioral problems.

Emergency care for MWDs with FB ingestion should be at immediate disposal because early surgical treatment may require fewer complex procedures and accelerate recovery [35]. One study revealed that gastric FB is a significant risk factor for GDV in dogs [49]. MWD breeds are predisposed to GDV, which is immediately life-threatening [9]. To prevent fatal GDV, preventing FB ingestion and performing prophylactic gastropexy are essential.

A limitation of this study is that medical data from the MWD training center or the ROKAF education and training command were not included. As AFMRI is a veterinary hospital for secondary care, some cases in which diagnosis and treatment were successfully provided by field unit veterinary officers might not be included in this study.

## 5. Conclusions

In conclusion, the clinical results of this study reveal that the most prevalent disease in the MWD group was GI disorder due to FB ingestion. To prevent ingestive behavioral problems, such as FB ingestion, coprophagy, anorexia, and hyperphagia, it is crucial to reduce the stress of MWDs by regularly improving their housing, living and working conditions, as well as maintaining a positive relationship with their handlers. For effective performance and good quality of life, MWDs with genetic factors of hip dysplasia should be excluded from breeding programs. The early detection and treatment of neurological diseases, such as DLSS and IVDD, in senior dogs through regular health checks are essential to increase their working lifespan and maintain good quality of life. Surgery for dental treatment and the removal of gastric FB (mainly leather collars or leashes) is generally performed at the AFMRI. Preventive care focusing on dental diseases and FB ingestion may be helpful in providing effective performance and good quality of life in MWDs.

## Figures and Tables

**Table 1:** Demographic summary of MWDs in this study.

Variables	Number	Percentage (%)
Duty		
Guard dog	239	67.71
Scout dog	80	22.66
Sniff dog	34	9.63
Breed		
German Shepherd	270	76.49
Belgian Malinois	45	12.75
Labrador Retriever	33	9.35
English Springer Spaniel	5	1.42
Sex		
Intact male	141	39.94
Intact female	24	6.8
Castrated male	74	20.96
Spayed female	114	32.39
Age group (years)		
Young adult (1 to 4)	140	39.66
Mature adult (5 to 9)	123	34.84
Senior (10 to 12)	80	22.66
End of life (≥13)	10	2.83

**Table 2:** The common disease categories of veterinary care among MWDs.

Category	Cases	Percentage (%)
Gastrointestinal	92	25.34
Dental	45	12.40
Musculoskeletal	44	12.12
Ear/Skin	40	11.02
Neurological	34	9.37
Oncological	26	7.16
Reproductive	24	6.61
Trauma	20	5.51
Hepatobiliary and exocrine pancreatic	17	4.68
Ophthalmological	7	1.93
Other ^a^	14	3.86
Total	363	100.00

^a^ Infectious (*n* = 4), respiratory (*n* = 3), cardiovascular (*n* = 2), endocrine (*n* = 2), hematological and immunological (*n* = 2), and urological (*n* = 1).

**Table 3:** Disease categories by breed.

Category	GSD		BM		LR	
	Cases	%	Cases	%	Cases	%
Gastrointestinal	69	23.88	18	39.13	5	17.86
Musculoskeletal	36	12.46	5	10.87	3	10.71
Neurological	33	11.42	0	0	1	3.57
Dental	31	10.73	7	15.22	7	25.00
Ear/Skin	30	10.38	3	6.52	7	25.00
Oncological	24	8.30	0	0	2	7.14
Reproductive	23	7.96	0	0	1	3.57
Hepatobiliary and exocrine pancreatic	15	5.19	2	4.35	0	0
Trauma	11	3.81	8	17.39	1	3.57
Ophthalmological	6	2.08	1	2.17	0	0
Infectious	3	1.04	1	2.17	0	0
Respiratory	2	0.69	0	0	1	3.57
Hematological and immunological	2	0.69	0	0	0	0
Endocrine	2	0.69	0	0	0	0
Urological	1	0.35	0	0	0	0
Cardiovascular	1	0.35	1	2.17	0	0
Total	289	100.00	46	100.00	28	100.00

Abbreviations: BM, Belgian Malinois; GSD, German Shepherd; LR, Labrador Retriever.

**Table 4:** Disease categories by age group.

Category	Young Adult		Mature Adult		Senior	
	Cases	%	Cases	%	Cases	%
Gastrointestinal	45	42.86	32	21.92	15	13.39
Musculoskeletal	17	16.19	16	10.96	11	9.82
Dental	10	9.52	26	17.81	9	8.04
Ear/Skin	9	8.57	20	13.70	11	9.82
Trauma	7	6.67	11	7.53	2	1.79
Reproductive	4	3.81	15	10.27	5	4.46
Hepatobiliary and exocrine pancreatic	3	2.86	7	4.79	7	6.25
Infectious	2	1.90	2	1.37	0	0
Neurological	2	1.90	5	3.42	27	24.11
Oncological	2	1.90	6	4.11	18	16.07
Urological	1	0.95	0	0	0	0
Hematological and immunological	1	0.95	0	0	1	0.89
Ophthalmological	1	0.95	3	2.05	3	2.68
Cardiovascular	1	0.95	1	0.68	0	0
Endocrine	0	0	1	0.68	1	0.89
Respiratory	0	0	1	0.68	2	1.79
Total	105	100.00	146	100.00	112	100.00

**Table 5:** Disease categories by season.

Category	Spring		Summer		Fall		Winter	
	Cases	%	Cases	%	Cases	%	Cases	%
Gastrointestinal	18	23.08	21	23.86	22	25.29	31	28.18
Ear/Skin	13	16.67	11	12.50	10	11.49	6	5.45
Neurological	11	14.10	6	6.82	6	6.90	11	10.00
Dental	11	14.10	9	10.23	11	12.64	14	12.73
Musculoskeletal	10	12.82	11	12.50	9	10.34	14	12.73
Reproductive	4	5.13	6	6.82	7	8.05	7	6.36
Oncological	4	5.13	3	3.41	7	8.05	12	10.91
Hepatobiliary and exocrine pancreatic	2	2.56	6	6.82	6	6.90	3	2.73
Trauma	2	2.56	6	6.82	5	5.75	7	6.36
Infectious	1	1.28	2	2.27	1	1.15	0	0
Endocrine	1	1.28	0	0	0	0	0	0
Respiratory	1	1.28	2	2.27	0	0	0	0
Ophthalmological	0	0	4	4.55	1	1.15	2	1.82
Hematological and immunological	0	0	1	1.14	0	0	1	0.91
Cardiovascular	0	0	0	0	2	2.30	0	0
Urological	0	0	0	0	0	0	1	0.91
Endocrine	0	0	0	0	0	0	1	0.91
Total	78	100.00	88	100.00	87	100.00	110	100.00

**Table 6:** Vector-borne pathogens in MWDs with positive SNAP^®^ 4Dx^®^ test.

Pathogen	Cases	Percentage (%)
*Anaplasma* spp.	28	56.00
*Dirofilaria immitis*	7	14.00
*Ehrlichia* spp.	6	12.00
*Borrelia burgdorferi* + *Ehrlichia* spp.	4	8.00
*Anaplasma* spp. + *Ehrlichia* spp.	3	6.00
*Anaplasma* spp. + *Borrelia burgdorferi*	1	2.00
*Borrelia burgdorferi*	1	2.00
Total	50	100.00

**Table 7:** The common surgical procedures.

Procedure	Cases	Percentage (%)
Dental scaling for periodontal disease	71	26.20
Tooth extraction	42	15.50
Upper GI endoscopy or gastrotomy for FB removal	42	15.50
Castration for male reproductive disease	25	9.23
Dental resin restoration	15	5.54
Repair of skin wound	14	5.17
Excision of gingival mass	13	4.80
Splenectomy	9	3.32
Repair of aural hematoma	8	2.95
Excision of skin mass	8	2.95
Other ^a^	24	8.86
Total	271	100.00

Abbreviations: FB, foreign body; GI, gastrointestinal. ^a^ Correction of gastric dilatation-volvulus (*n* = 3), enteroenteropexy (*n* = 3), dorsal laminectomy (*n* = 2), enterotomy (*n* = 2), sialadenectomy (*n* = 2), caudectomy (*n* = 1), cholecystectomy (*n* = 1), enucleation (*n* = 1), excision of mesenteric mass (*n* = 1), exploratory laparotomy (*n* = 1), gingival flap (*n* = 1), hemilaminectomy (*n* = 1), intestinal resection and anastomosis (*n* = 1), omental flap (*n* = 1), repair of eyelid laceration (*n* = 1), root canal therapy (*n* = 1), and unilateral arytenoid lateralization (*n* = 1).

**Table 8:** Definitive diagnoses for MWDs with gait abnormality.

Diagnosis	Cases	Percentage (%)
Musculoskeletal	47	75.81
Degenerative joint disease	26	41.94
Sprain/Strain	17	27.42
Cranial cruciate ligament disease	2	3.23
Immune-mediated myositis	1	1.61
Carpal hyperextension	1	1.61
Neurological	10	16.13
Intervertebral disc disease	7	11.29
Degenerative lumbosacral stenosis	3	4.84
Traumatic		
Foot pad injury	3	4.84
Reproductive		
Orchitis	1	1.61
Oncological		
Prostatic adenocarcinoma	1	1.61
Total	62	100.00

**Table 9:** Types of foreign objects.

Type	Cases	Percentage (%)
Leather collar or leash	9	29.03
Metallic objects	5	16.13
Towels or gloves	5	16.13
Plastic objects	3	9.68
Stones and sand	2	6.45
Ball	2	6.45
String	2	6.45
Oxygen absorber	1	3.23
Fruit seeds	1	3.23
Electric wires	1	3.23
Total	31	100.00

**Table 10:** The locations of foreign objects and methods for removal.

Category	Cases	Percentage (%)
Location		
Esophagus	1	1.92
Stomach	41	78.85
Small intestine	6	11.54
Large intestine	4	7.69
Removed by		
Upper GI endoscopy	26	50.00
Gastrotomy	16	30.77
Spontaneous evacuation	8	15.38
Enterotomy	2	3.85
Total	52	100.00

Abbreviation: GI, gastrointestinal.

## Data Availability

Not applicable.
